# Exploring Serum Biomarkers for Neuropathic Pain in Rat Models of Chemotherapy-Induced Peripheral Neuropathy: A Comparative Pilot Study with Oxaliplatin, Paclitaxel, Bortezomib, and Vincristine

**DOI:** 10.3390/toxics11121004

**Published:** 2023-12-08

**Authors:** David Balayssac, Julie Durif, Céline Lambert, Cristelle Dalbos, Eric Chapuy, Monique Etienne, Claire Demiot, Jérôme Busserolles, Vincent Martin, Vincent Sapin

**Affiliations:** 1 Direction de la Recherche Clinique et de l’Innovation, CHU Clermont-Ferrand, F-63000 Clermont-Ferrand, France; 2 INSERM U1107 NEURO-DOL, Université Clermont Auvergne, F-63000 Clermont-Ferrand, France; cristelle.dalbos@uca.fr (C.D.); eric.chapuy@uca.fr (E.C.); jerome.busserolles@uca.fr (J.B.); 3 Laboratoire de Biochimie et de Génétique Moléculaire, CHU Clermont-Ferrand, F-63000 Clermont-Ferrand, France; j_durif@chu-clermontferrand.fr; 4 Unité de Biostatistiques, Direction de la Recherche Clinique et de l’Innovation, CHU Clermont-Ferrand, F-63000 Clermont-Ferrand, France; clambert@chu-clermontferrand.fr; 5 Metabolic Adaptations to Exercise under Physiological and Pathological Conditions (AME2P), Université Clermont Auvergne, F-63000 Clermont-Ferrand, France; monique.etienne@uca.fr (M.E.);; 6 UR 20218—Neuropathies et Innovations Thérapeutiques (NeurIT), Faculties of Medicine and Pharmacy, University of Limoges, F-87025 Limoges, France; claire.demiot@unilim.fr; 7 Institut Universitaire de France (IUF), F-75000 Paris, France; 8 Laboratoire de Biochimie et de Génétique Moléculaire, CNRS, INSERM, iGReD, CHU Clermont-Ferrand, Université Clermont Auvergne, F-63000 Clermont-Ferrand, France; vsapin@chu-clermontferrand.fr

**Keywords:** chemotherapy-induced peripheral neuropathy, oxaliplatin, paclitaxel, bortezomib, vincristine, animal model, neuropathic pain, blood biomarker

## Abstract

Blood biomarkers, including neurofilament light chain (NfL), have garnered attention as potential indicators for chemotherapy-induced peripheral neuropathy (CIPN), a dose-limiting adverse effect of neurotoxic anticancer drugs. However, no blood biomarker has been established for routine application or translational research. This pilot study aimed to evaluate a limited panel of blood biomarkers in rat models of CIPN and their correlations with neuropathic pain. CIPN models were induced through repeated injections of oxaliplatin, paclitaxel, bortezomib, and vincristine. Electronic von Frey testing was used to assess tactile allodynia. Post anticancer injections, serum concentrations of 31 proteins were measured. Allodynia thresholds decreased in anticancer-treated animals compared to controls. No consistent modifications were observed in the biomarkers across CIPN models. The most noteworthy biomarkers with increased concentrations in at least two CIPN models were NfL (paclitaxel, vincristine), MCP-1, and RANTES (oxaliplatin, vincristine). Vincristine-treated animals exhibited strong correlations between LIX, MCP-1, NfL, and VEGF concentrations and tactile allodynia thresholds. No single biomarker can be recommended as a unique indicator of CIPN-related pain. Because of the study limitations (single dose of each anticancer drug, young animals, and single time measurement of biomarkers), further investigations are necessary to define the kinetics, specificities, and sensitivities of MCP-1, RANTES, and NfL.

## 1. Introduction

Chemotherapy-induced peripheral neuropathy (CIPN) is a common adverse effect of neurotoxic anticancer drugs, including platinum derivatives (cisplatin, oxaliplatin), spindle poisons (taxanes: paclitaxel, docetaxel; vinca alkaloids: vincristine; epothilones; eribulin), bortezomib, and thalidomide [1]. CIPN is typically described as a distal and symmetric polyneuropathy with a “stockings and gloves” distribution. Its overall symptomatology includes paresthesia (tingling, numbness) and dysesthesia (thermal and tactile allodynia, and neuropathic pain). The prevalence of CIPN is approximately 68.1% in the first month after the end of chemotherapy, 60.0% at three months, and 30.0% after six months [2]. This condition significantly affects health-related quality of life and causes psychological distress [3,4]. Currently, no preventive strategy is recommended, and duloxetine has a moderate recommendation for the management of pain from the American Society of Clinical Oncology (ASCO) and the European Society for Medical Oncology (ESMO) for managing neuropathic pain [5,6]. Consequently, oncologists may need to consider reducing or discontinuing the neurotoxic anticancer regimen to mitigate CIPN severity [5,6,7], even though this approach may negatively impact disease control and progression-free survival [8].

CIPN screening in patients lacks a gold standard. A combination of clinician-reported outcome measures (i.e., clinical examination, electrophysiological testing, quantitative sensory testing) and patient-reported outcome measures (self-administered questionnaires) would be the best option [6]. Unfortunately, these examinations are time-consuming during oncological follow-up [9]. In patients at risk of serious neurotoxicity, more extensive CIPN evaluations remain critical [10]. Given that CIPN severity is linked to the dose of neurotoxic anticancer drugs [6], identifying sensitive and specific biomarkers would enable clinicians to modify anticancer protocols and limit its occurrence and severity.

Circulating blood biomarkers of neurotoxicity, such as neurofilament light chain (NfL), have shown promise for CIPN screening in both animal models (cisplatin or paclitaxel) [11,12] and humans (paclitaxel and carboplatin [13]; oxaliplatin [14]). However, despite being viewed as an objective and innovative strategy for detecting CIPN, no single biomarker has yet proven useful for diagnosing and monitoring CIPN, and routine application is not recommended according to the ESMO [6].

CIPN is a unique terminology used to define the symptomatology of the peripheral neurotoxicity of anticancer drugs. But, CIPN results from the neurotoxic effect of different anticancer drugs with different toxicodynamic pathways (platinum adducts to nucleic acids for platinum derivatives, alteration of microtubules turnover for spindle poisons, and inhibition of the 26S proteasome for bortezomib). Besides ion channel disorders, oxidative stress, and neuroinflammation, no clear unique pathways can be defined for all the neurotoxic anticancer drugs [15]. In the same way, symptomatology is grossly the same across different neurotoxic anticancer drugs [15], but differences exist regarding thermal sensitivity. For example, in cancer patients, oxaliplatin is responsible for cold and heat-triggered pain, without alteration of cool and warm detection thresholds [16]. Cisplatin is not responsible for any thermal alteration [16]. Bortezomib is responsible for a loss of heat pain perception and warm detection thresholds, without alteration of cold pain and cool detection thresholds [17]. Vincristine is responsible for a loss of perception for cold and heat pain, as well as cool and warm detection [18].

Consequently, it is important to keep in mind that these blood biomarkers for CIPN should be sufficiently robust to be usable for any neurotoxic anticancer drug, encompassing all these neurotoxic mechanisms. Moreover, these blood biomarkers should be validated in animal models of CIPN, enhancing the translationality of non-clinical studies in rodents and improving the predictive validity of pathophysiological and pharmacological findings.

The objective of this pilot study was to evaluate a panel of blood biomarkers in four animal models of CIPN and investigate their relationship with neuropathic pain. The selection of blood biomarkers was based on the existing scientific literature focused on CIPN or peripheral neuropathy. These biomarkers include NfL, which exhibits increased blood concentrations in response to oxaliplatin treatment in colorectal cancer patients and is associated with the severity grade of CIPN [14]; glial fibrillary acidic protein (GFAP), demonstrating elevated blood concentrations in patients suffering from chronic sensory-motor axonal neuropathy, chronic inflammatory demyelinating polyneuropathy, and multifocal motor neuropathy [19]; osteopontin (OPN), showing an inverse correlation between blood concentrations assessed before taxane-based chemotherapy and axonal loss in the sural nerve after treatment [20]; and nerve growth factor (NGF), displaying increased blood concentrations in cancer patients with painful CIPN [21]. In addition, we utilized available biomonitoring kits to explore pro- and anti-inflammatory cytokines (Milliplex^®^, Rat Cytokine/Chemokine Panel—27 Plex, Merck, Saint Quentin en Yvelines, France) due to the involvement of neuroinflammatory processes in animal models of CIPN. This includes an increase in blood concentrations of tumor necrosis factor alpha (TNFα), interferon gamma (IFNγ), interleukin 1 alpha (IL-1a), interleukin 1 beta (IL-1b), interleukin 2 (IL-2), interleukin 6 (IL-6), and monocyte chemoattractant protein 1 (MCP-1) in patients or rodents treated with neurotoxic anticancer drugs [22,23]. The chosen animal models of CIPN have been previously documented in the literature for investigating CIPN pathophysiology and identifying potential pharmacological targets [24]. Neuropathic pain was selected as the primary outcome measure due to its convenience and non-invasiveness for assessing peripheral neuropathy in animals. Additionally, it is characteristic of CIPN severity in patients [3,4].

## 2. Materials and Methods

### 2.1. Animals

Experiments were performed with 96 animals (five-week-old male Sprague Dawley rats, Janvier Labs, Saint Berthevin, France). Twenty-four rats were allocated for each animal model of CIPN (twelve control and twelve anticancer drug-treated animals). Animals were housed (four per cage) in the animal facility, with water and food ad libitum, and kept in conditions of 12:12 h light/dark cycle (non-reversed) and 50% hygrometry. Animals were acclimated to the animal facility for five days before the beginning of the experiments. All the experiments were conducted in accordance with the ARRIVE guideline [25]. The present study received ethical approval from the Animal Care and Use Committee of Auvergne (C2EA-02) and from the French Ministry of Higher Education, Research and Innovation (Ministère de l’Enseignement supérieur, de la Recherche et de l’Innovation), with the following agreement number: N°APAFIS#21686. The number of experimental animals was kept to a minimum.

### 2.2. Animal Models of Chemotherapy-Induced Peripheral Neuropathy

**Figure 1:**
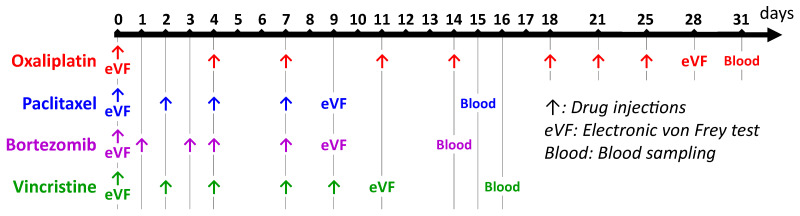
Timeline for the induction of animal models of CIPN, assessment of tactile allodynia, and blood sampling.

Oxaliplatin (Leancare, Conwy, UK) was administered intravenously eight times (2 mg/kg) on days 0, 4, 7, 11, 14, 18, 21, and 25, resulting in a cumulative dose of 16 mg/kg (Figure 1). For each injection, oxaliplatin was diluted to 2 mg/mL in a 5% glucose solution from a 4 mg/mL stock solution (solvent: 5% glucose) [26]. Control animals received the same volume of the vehicle (5% glucose).

Paclitaxel (Leancare, Conwy, UK) was injected intraperitoneally four times (2 mg/kg) on days 0, 2, 4, and 7, resulting in a cumulative dose of 8 mg/kg (Figure 1). For each injection, paclitaxel was diluted to 1 mg/mL in 0.9% NaCl from a 6 mg/mL stock solution (solvent: cremophor^®^EL (C5135, Sigma-Aldrich, Saint-Louis, France)/ethanol, 1/1) [27]. Control animals received the same volume of the vehicle (cremophor^®^EL/ethanol (1/1) diluted 1/6 in 0.9% NaCl).

Bortezomib (Leancare, Conwy, UK) was injected intraperitoneally five times (0.2 mg/kg) on days 0, 1, 3, 4, and 7, resulting in a cumulative dose of 1 mg/kg (Figure 1). For each injection, bortezomib was diluted to 0.1 mg/mL in 0.9% NaCl (vehicle) from a 1 mg/mL stock solution (solvent: 5% DMSO (276855, Sigma-Aldrich, Saint-Louis, France)) (adapted from Yamamoto et al. [28]). Control animals received the same volume of the vehicle (5% DMSO diluted 1/10 in 0.9% NaCl).

Vincristine (Leancare, Conwy, UK) was injected intravenously five times (0.15 mg/kg) on days 0, 2, 4, 7, and 9, resulting in a cumulative dose of 0.75 mg/kg (Figure 1). For each injection, vincristine was diluted to 0.15 mg/mL in 0.9% NaCl (vehicle) from a 1 mg/mL stock solution (solvent: 0.9% NaCl) (adapted from [29]). Control animals received the same volume of the vehicle (0.9% NaCl).

To prevent anticancer drug exposure between animals within the same cage through feces and urine, treatments for each animal model were randomized by the entire cage of animals.

### 2.3. Assessment of Nociceptive Disorders (Tactile Allodynia)

Tactile nociceptive thresholds were assessed using an electronic von Frey test (Bioseb, Vitrolles, France) (Figure 1) [30]. Tactile allodynia is a common sensory disorder reported in animal models of CIPN, whereas thermal disorders are less consistently observed between CIPN models [24,31].

Rats were placed individually in plastic compartments on an elevated wire floor and allowed to habituate for 15 min before each experiment. The von Frey apparatus, consisting of a plastic tip fitted in a hand-held force transducer, was applied perpendicularly to the animal’s right hind paw from below, and the force applied was gradually increased until paw withdrawal. The maximum force applied (expressed in grams) to induce paw withdrawal was recorded automatically. The two measurements not differing by more than 10 g were averaged and assigned as the nociceptive threshold [30]. Three days before CIPN induction, each rat was habituated to the plastic compartments to avoid any fear or stress during the experiment. The experimenter was blinded to the treatment attribution.

### 2.4. Assessments of Serum Biomarkers

Blood samples were collected approximately one week after the last injection of anticancer drugs in order to avoid unexpected interferences between biomarkers and anticancer drugs (Figure 1). The tactile allodynia induced by these anticancer drugs has been described to be stable several days after the last injection of these anticancer drugs [31,32]. Blood was collected in vials with clot activator and kept at room temperature for a maximum of one hour. Thereafter, blood samples were centrifugated at 4100 rpm at 20 °C for 10 min. Finally, serum was collected, aliquoted, and stored at −80 °C until analysis.

Concentrations of 31 biomarkers were assessed in serum, using available commercial kits, and according to the manufacturer’s instructions (Supplementary file Table S1). For all the analytes measured, serum samples were randomized on assay plates to reduce possible batch effects. When the serum concentration of a biomarker was not quantifiable and below the lower limit of quantification, the value of the concentration was replaced by the lower limit of quantification of the biomarker [33].

### 2.5. Statistical Analysis

Statistical analysis was performed using Stata (version 15, StataCorp, College Station, TX, USA) and R (version 4.1.3; R Foundation for Statistical Computing, Vienna, Austria). All the tests were two-sided, with an alpha level set at 5%. All the continuous variables were expressed by mean ± standard deviation. Comparisons of repeated continuous variables (electronic von Frey test and weight of animals) were performed using a repeated-measure ANOVA and followed by a post hoc Tukey–Kramer test. The serum concentrations of biomarkers of control animals were compared according to the four anticancer drugs using the Kruskal–Wallis test. The serum concentrations of biomarkers were compared between treated and control animals using the Mann–Whitney test. The results were expressed as effect sizes (ES) with their 95% confidence interval (95%CI), and interpreted according to Cohen’s recommendations [34]: 0.2 = small effect, 0.5 = medium effect, and 0.8 = large effect. Spearman’s correlation coefficients were calculated between serum concentrations of biomarkers and tactile allodynia thresholds assessed after the end of anticancer drug injections and interpreted as follows (absolute value): ≥0.70 = strong correlation, 0.50–0.69 = moderate correlation, 0.30–0.49 = low correlation, 0.00–0.29 = no or negligible correlation. Finally, a factor analysis of mixed data was performed to study the similarities between animals, considering both continuous and categorical variables, as well as to examine the relationships among all variables. For this analysis, variables were chosen according to clinical relevance and statistical distribution (characteristics always present or always absent were not considered), and only animals without missing data were used (n = 70).

## 3. Results

### 3.1. Animal Models of CIPN

**Figure 2:**
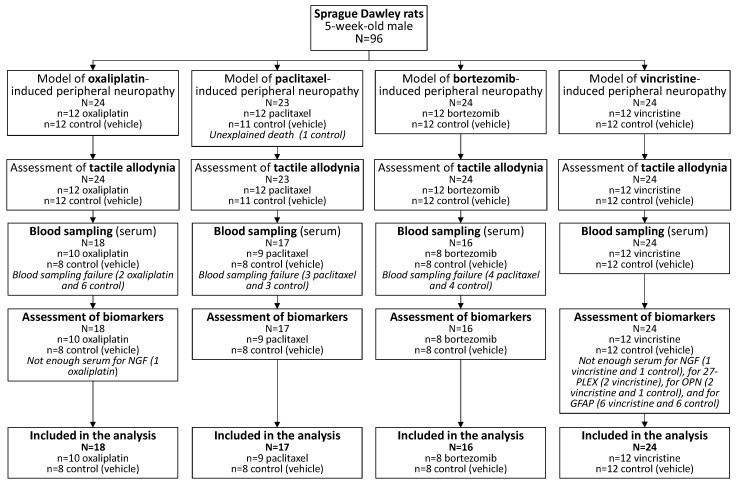
Flow diagram of animal inclusion in the analysis.

The flow chart of the animals analyzed is presented in Figure 2.

**Figure 3:**
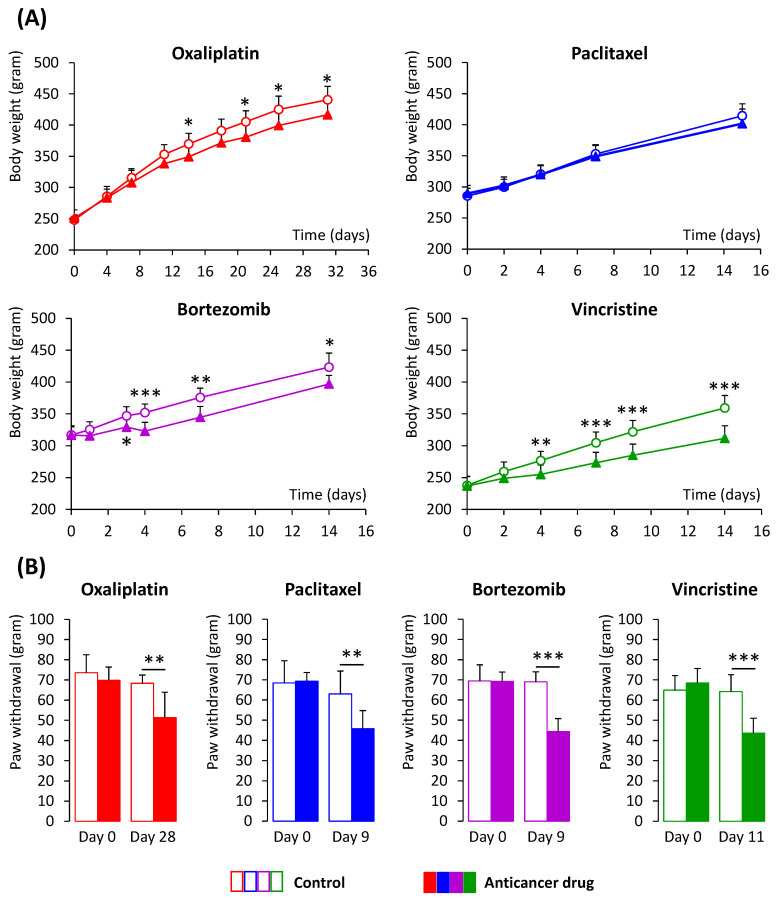
Weight of animals and tactile allodynia thresholds. (**A**) Weight of animals treated by anticancer drugs (oxaliplatin, paclitaxel, bortezomib, and vincristine) in comparison to control animals. The results are expressed in grams and presented by mean + standard deviation for control animals (white circle, n = 8–12 animals per anticancer drugs) and anticancer-treated animals (full triangle, n = 8–12 animals per anticancer drugs). Black arrows indicate the day of the anticancer drug injection. (**B**) Tactile allodynia thresholds (electronic von Frey test) of animals treated by anticancer drugs (oxaliplatin, paclitaxel, bortezomib, and vincristine) in comparison to control animals, before (day 0—basal values) and after the end of anticancer drug injections. The results are expressed in grams and presented by mean + standard deviation for control animals (white bar, n = 8–12 animals per group) and anticancer-treated animals (full bar, n = 8–12 animals per group). * *p* < 0.05, ** *p* < 0.01, and *** *p* < 0.001 control vs. anticancer drug treated animals (repeated-measure ANOVA followed by a post hoc Tukey–Kramer test).

Oxaliplatin-, bortezomib-, and vincristine-treated animals (not paclitaxel-treated animals) presented a lower body weight than the control animals starting from day 14 for oxaliplatin, day 3 for bortezomib, and day 4 for vincristine, and until the end of the experiment (Figure 3A).

At the end of the anticancer drug injections, tactile allodynia thresholds of anticancer drug-treated animals were lower than control ones (ES and 95%CI for oxaliplatin: −1.65 [−2.68 to −0.58]; paclitaxel: −1.61 [−2.66 to −0.52]; bortezomib: −4.08 [−5.82 to −2.30]; vincristine: −2.50 [−3.55 to −1.42]). No difference of tactile allodynia thresholds between the anticancer-treated animals and control ones was reported on day 0 (basal value) (Figure 3B).

### 3.2. Serum Biomarkers of Neuropathic Pain

Concentrations of several biomarkers were significantly different between control animals among the four animal models of CIPN (Fractalkine: *p* = 0.01, IL-1α: *p* = 0.04, IL-6: *p* = 0.04, IL-13: *p* = 0.01, IL-17A: *p* = 0.01, LIX: *p* = 0.02, and VEGF: *p* = 0.02 (Table 1)). In most cases, control animals of the paclitaxel model had higher concentrations of these biomarkers than control animals of other CIPN models (except for LIX, for which control animals of oxaliplatin and paclitaxel had lower concentrations than control animals of other CIPN models). Because of these variations in biomarkers in control animals, it was decided not to pool the results of biomarker concentrations for control animals in order to perform a comparison between a single control group and each anticancer drug.

**Figure 4:**
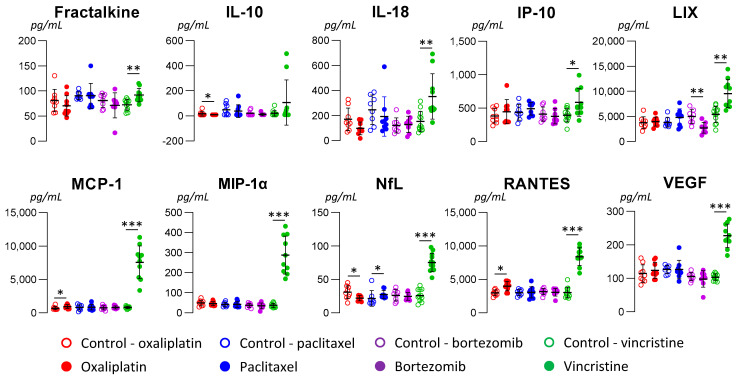
Concentrations of the most significant biomarkers (Fractalkine, IL-10, IL-18, IP-10, LIX, MCP-1, MIP-1α, NfL, RANTES, and VEGF). Results are presented by the mean ± standard deviation for each biomarker and for each animal group. * *p* < 0.05, ** *p* < 0.01, and *** *p* < 0.001 (Kruskal–Wallis test).

Comparisons of biomarker concentrations between anticancer-treated animals and control ones are presented in Figure 4 (details of all the biomarker concentrations, see Supplementary Table S1). 

For oxaliplatin, the MCP-1 and RANTES concentrations were significantly higher in anticancer drug-treated animals than in control ones (ES and 95%CI: 1.42 [0.39 to 2.41] *p* = 0.01, and 1.29 [0.28 to 2.26] *p* = 0.02, respectively). NfL concentrations were significantly lower in anticancer drug-treated animals than in control ones (−1.36 [−2.34 to −0.34] *p* = 0.01). IL-10 concentration was lower in oxaliplatin-treated animals (*p* = 0.04), but the effect size was not significant (−0.71 [−1.62 to 0.22]).

For paclitaxel, only the NfL concentration tended to be higher in anticancer drug-treated animals than in control ones (ES and 95%CI: 0.63 [−0.31 to 1.55] *p* = 0.03).

For bortezomib, only the LIX concentration was significantly lower in anticancer drug-treated animals than in control ones (ES and 95%CI: −1.72 [−2.82 to −0.58] *p* = 0.003).

For vincristine, fractalkine, IL-18, IP-10, LIX, MCP-1, MIP-1α, NfL, RANTES, and VEGF concentrations were significantly higher in anticancer drug-treated animals than in control ones (ES and 95%CI: 1.56 [0.60 to 2.48] *p* = 0.002, 1.42 [0.49 to 2.33] *p* = 0.003, 1.17 [0.27 to 2.04] *p* = 0.01, 1.79 [0.80 to 2.75] *p* = 0.001, 3.93 [2.46 to 5.37] *p* < 0.001, 3.77 [2.34 to 5.17] *p* < 0.001, 4.15 [2.69 to 5.58] *p* < 0.001, 4.88 [3.16 to 6.57] *p* < 0.001, and 4.79 [3.10 to 6.46] *p* < 0.001, respectively). It is to be noticed that the OPN concentration was higher in bortezomib-treated animals (*p* = 0.03), but the effect size was not significant (0.71 [−0.15; 1.55]).

Finally, none of the variations in biomarker concentrations (increase or decrease) were similar between animal models of CIPN. Only the MCP-1 and RANTES concentrations showed a similar increase both in oxaliplatin- and vincristine-treated animals; likewise for NfL concentrations for paclitaxel- and vincristine-treated animals. Moreover, LIX concentrations presented opposite variations between bortezomib- and vincristine-treated animals, and similarly for NfL concentrations for oxaliplatin- and for both paclitaxel- and vincristine-treated animals.

**Figure 5:**
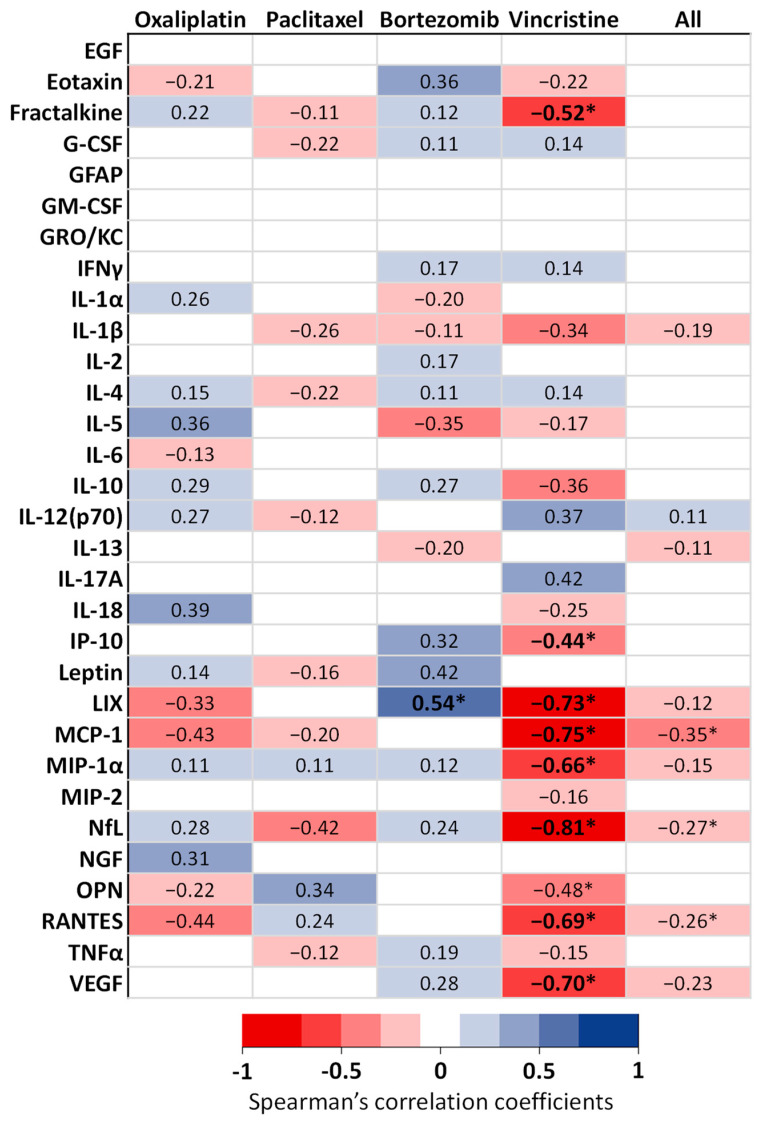
Heatmap of the correlations between the serum concentrations of biomarkers and tactile allodynia thresholds after the end of anticancer injections, and for each anticancer drug. Correlation coefficients were calculated between tactile allodynia thresholds on day 28 for oxaliplatin, on day 9 for paclitaxel, on day 9 for bortezomib, and on day 11 for vincristine, and biomarker concentrations, including all the animals (controls and anticancer drug-treated animals). * *p* < 0.05.

Correlations between biomarker concentrations and tactile allodynia thresholds assessed after the end of anticancer drug injections, and for all animals (control + anticancer drug-treated animals), are presented in Figure 5. For oxaliplatin and paclitaxel models, no significant correlation was reported between biomarker concentrations and tactile allodynia thresholds. For the bortezomib model, a moderate positive correlation was reported between LIX concentrations and tactile allodynia thresholds, indicating that the biomarker concentration increases as the tactile allodynia thresholds increase. For the vincristine model, strong negative correlations were reported between LIX, MCP-1, NfL, and VEGF concentrations and tactile allodynia thresholds, indicating that the biomarkers’ concentrations increase as the tactile allodynia thresholds decrease. Additionally, moderate negative correlations were observed for fractalkine, MIP-1α, and RANTES concentrations. When pooling all the animal models, only the MCP-1 concentrations were significantly and negatively correlated (low level) with tactile allodynia thresholds. The NfL and RANTES concentrations were significantly and negatively correlated with tactile allodynia thresholds (negligible level).

**Figure 6:**
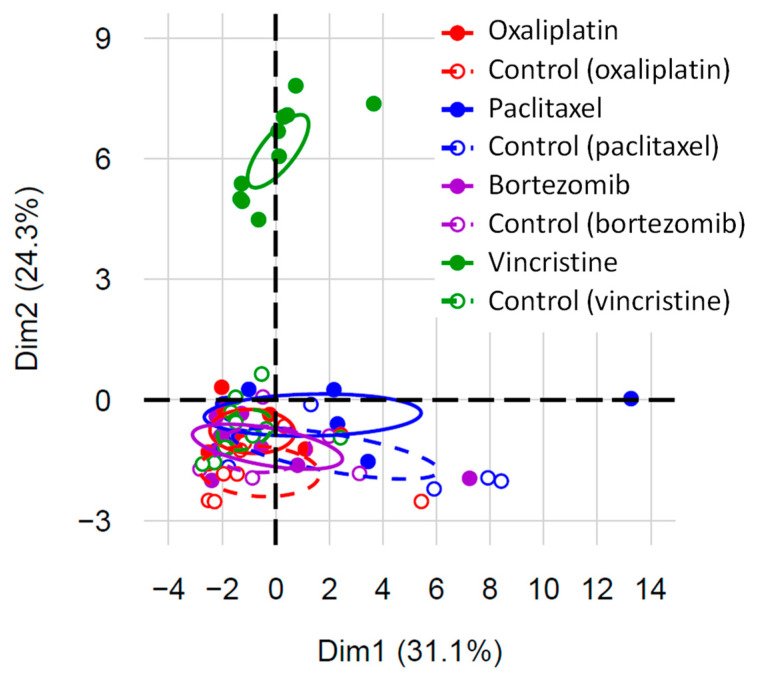
Two-dimensional representation of the factorial analysis of mixed data. The factorial analysis of mixed data included continuous variables (last weight measures, variations in weight between the first and the last measures, last tactile allodynia thresholds, variations in tactile allodynia threshold between the first and the last measures, serum concentrations of fractalkine, IL-1β, IL-2, IL-12(p70), IL-5, IL-17A, IL-18, IP-10, leptin, LIX, MIP-1α, MCP-1, NfL, OPN, RANTES, VEGF, and TNFα) and categorical variables (serum concentrations of eotaxin (≤4.65, >4.65), G-CSF (≤4.48, >4.48), IFNγ (≤10.45, >10.45), IL-1α (≤39.845, >39.845), IL-4 (≤17.93, >17.93), IL-6 (≤279.85, >279.85), IL-13 (≤17.525, >17.525), and IL-10 (≤8.27, >8.27)). The treatment group (oxaliplatin-treated, oxaliplatin vehicle-treated (control of oxaliplatin), paclitaxel-treated, paclitaxel vehicle-treated (control of paclitaxel), bortezomib-treated, bortezomib vehicle-treated (control of bortezomib), vincristine-treated, and vincristine vehicle-treated (control of vincristine)) was added as a supplementary variable. The following variables were not included in the model: serum concentrations of EGF, GFAP, GM-CSF, GRO/KC, MIP-2, and NGF.

A factorial analysis of mixed data was conducted on selected quantitative and qualitative variables (for details, see Figure 6) to explore the relationship between different variables to determine whether they have an impact on each other and, here, to explore whether the concentrations of biomarkers would aid in discriminating CIPN models. The model used was able to report data in a two-dimensional plot diagram (Figure 6). In this analysis, all the vincristine-treated animals (green full dots, Figure 6) were isolated from other animals (control and anticancer drug-treated animals), whereas nearly all the animals (control and anticancer-treated animals) of other animal models were plotted together. Interestingly, the three control animals of the paclitaxel model tended to be isolated from other animals (observation to be interpreted with caution).

## 4. Discussion

The aim of this study was to assess a panel of serum proteins as biomarkers of neuropathic pain in validated animal models of CIPN [24,35] to identify the most relevant biomarkers and candidates for further non-clinical and translational studies.

No serum biomarkers assessed showed consistent modifications across all four animal models of CIPN used. Consequently, none of these biomarkers can be recommended as a unique circulating biomarker of CIPN-related neuropathic pain, regardless of the anticancer drug involved. The most interesting biomarker would be one that could be used for different neurotoxic anticancer drugs. However, some of the assessed biomarkers whose serum concentrations were consistently modified in at least two CIPN models (anticancer drug vs. control) could be of interest, such as NfL (paclitaxel and vincristine), MCP-1 (oxaliplatin and vincristine), and RANTES (oxaliplatin and vincristine).

NfL is probably the most frequently studied circulating biomarker of CIPN. Blood concentrations of NfL and other neurofilaments are typically interpreted as markers of axonal damage, a common feature across various neurological diseases characterized by demyelination, neuropathy, dendritic loss, cell death, and chronic inflammation [36]. In rats, serum NfL levels rapidly increased after administrations of paclitaxel (10 mg/kg, intravenous injections once a week for 4 weeks), cisplatin (2 mg/kg, intraperitoneal injections twice a week for 4 weeks), and vincristine (0.2 mg/kg, intravenous injection once/week for 4 weeks). For these three animal models of CIPN, serum NfL concentrations significantly increased from the first week of treatment until the end of the experiments, compared to control animals. Peripheral neuropathy was confirmed by morphological and neurophysiological alterations in caudal nerves [11,37]. In mice, two studies identified an increase in blood NfL concentrations in animals treated by repeated injections of paclitaxel (15 mg/kg, 7 injections for 15 days [12]; 2 mg/kg, 4 injections for 8 days [38]). However, one study did not find any increase in blood NfL concentrations after repeated injections of oxaliplatin (9 mg/kg, 6 injections for 19 days [12]). In patients, serum NfL concentrations have been associated with oxaliplatin-related CIPN in colorectal cancer patients and associated with CIPN grade and electrophysiological disorders [14]. Similarly, serum NfL concentrations have been associated with paclitaxel ± carboplatin-related CIPN in breast, ovarian, or endometrial cancer patients and correlated with the scores of CIPN questionnaires [39,40,41], CIPN grade [13,40,42], and nerve conduction disorders [41,43]. Finally, serum NfL concentrations were higher in multiple myeloma patients with an ongoing bortezomib treatment than multiple myeloma patients after past bortezomib treatment and healthy controls [44]. In this study, serum NfL concentrations were correlated with electrophysiological disorders. Interestingly, serum NfL concentrations were not associated with pain [44].

MCP-1, also known as C-C motif ligand 2 (CCL2), has been described as a biomarker of neuroinflammation, and its concentrations in blood and cerebrospinal fluid have been found to increase in patients with various neurological diseases, including Parkinson’s disease, multiple sclerosis, ischemic stroke, and traumatic brain injury [45,46]. Plasma concentrations of MCP-1 have been associated with pain severity in fibromyalgia patients but not associated with other covariates such as body mass index, medications, severity of depression, and overall fibromyalgia burden [47]. Recently, it has been suggested that MCP-1 could serve as a severity marker of the neuropathy in patients [48].

RANTES, also known as C-C motif ligand 5 (CCL5), is a pro-inflammatory chemokine involved in regulating immunoreactions and recruiting immune cells, such as monocytes, granulocytes, and T cells, to sites of inflammation [49]. Blood concentrations of RANTES have also been associated with neurological diseases, including Parkinson’s disease, traumatic brain injury, and human immunodeficiency peripheral neuropathy [45,50,51].

Both serum concentrations of MCP-1 and RANTES were increased in the same animal models of CIPN (oxaliplatin and vincristine), suggesting a potential shared neuroinflammatory pathway. It is worth noting that neuroinflammation has been described in all types of CIPN [52,53].

In the animal model of vincristine-induced peripheral neuropathy, several concentrations of biomarkers were strongly (LIX, MCP-1, NfL, and VEGF) to moderately (fractalkine, MIP-1α, and RANTES) correlated with tactile allodynia thresholds, whereas for the bortezomib model, only LIX concentrations were moderately correlated with tactile allodynia thresholds, and no such correlations were reported for oxaliplatin and paclitaxel. Moreover, the factorial analysis of mixed data performed on all the study data (including the concentrations of biomarkers) discriminated vincristine-treated animals from all the other ones (treated and control animals from other animal models of CIPN). No clear explanation can be proposed. Some subtle differences in pathological pathways have been identified in bioengineered sensory nerve tissue exposed to vincristine, cisplatin, or paclitaxel [54], as well as in animal models of paclitaxel and oxaliplatin [55]. However, all these neurotoxic anticancer drugs share common pathological pathways, including oxidative stress, apoptosis, changes in cellular calcium homeostasis, axonal damage, alterations in neuronal excitability, immune system activation, and inflammation of nervous tissues [53]. In the clinical setting, differences among neurotoxic anticancer drugs remain poorly explored. For example, both oxaliplatin- and paclitaxel-related CIPN are characterized by acute symptoms and a worsening of CIPN with the repetition of chemotherapy cycles. However, symptoms improve after paclitaxel cessation and worsen after oxaliplatin treatment [56]. Another example of differences in symptoms between neurotoxic anticancer drugs can be observed in quantitative sensory testing. Oxaliplatin is known to induce cold and heat hypersensitivity [16], while bortezomib induces heat hyposensitivity [17].

Control animals in the paclitaxel model exhibited different serum biomarker concentrations compared to control animals in other CIPN models. This difference suggests that the paclitaxel vehicle (1/6 diluted Cremophor^®^EL/ethanol 50/50) may impact these serum biomarker concentrations. Cremophor^®^EL has previously been identified as a potential neurotoxic agent in rats, leading to mechanical hyperalgesia and allodynia, which are associated with peripheral axonal damage [57].

The primary outcome used to assess and validate CIPN in animals was the presence of nociceptive disorders, specifically tactile allodynia thresholds assessed using the electronic von Frey test. Neuropathic pain serves as a reliable marker of CIPN severity in patients [3,4,58]. Measuring nociceptive thresholds in living rodents is convenient and allows for repeatability [59]. In this study, it might have been possible to enhance nociceptive threshold assessment with additional behavioral tests, such as thermal sensitivity. However, it is worth noting that thermal sensitivity is not consistently reported in all animal models of CIPN. For instance, in rat models of bortezomib-, oxaliplatin-, paclitaxel-, or vincristine-related CIPN, cold allodynia was observed, but only bortezomib, oxaliplatin, and paclitaxel models exhibited heat allodynia [26,31]. While histopathological analyses of peripheral nervous system tissues and electrophysiological analyses of peripheral nerves could be used to validate CIPN in animals [60,61], they are often considered cumbersome, diverge from clinical routine practices [62,63], and may limit finally the translationality of studies.

To further explore and validate the potential translational implications of our study findings, it is essential to investigate NfL, MCP-1, and RANTES in cancer patients receiving neurotoxic anticancer drugs, particularly in relation to the occurrence of CIPN. As of now, NfL stands out as the most extensively studied biomarker for CIPN. A translational study has highlighted its good correlation with paclitaxel neurotoxicity, both in vitro using induced pluripotent stem cell-derived sensory neurons and in cancer patients [39]. Recently, another clinical study corroborated the good correlation between blood concentrations of NfL and the severity of CIPN in breast cancer patients treated with paclitaxel. NfL levels showed a strong correlation with cumulative doses of paclitaxel, moderate correlations with sensory scores from the QLQ-CIPN20 (self-administered questionnaire on CIPN), and total neuropathy scores (clinician-reported outcome) [64]. Although more data are required to fully validate the utility of NfL, it has the potential to serve as a biomarker for monitoring and mitigating CIPN.

However, the relationship between blood concentrations of MCP-1 and RANTES with CIPN severity has not been explored in any clinical assay to date. Initiating these studies in cancer patients is crucial.

The cornerstone of these cross-validations between in vitro/in vivo studies and clinical assays requires an assessment for each class of neurotoxic anticancer drugs (i.e., platinum derivatives, taxanes, vinca alkaloids, and bortezomib) to ensure the robustness of the biomarker. In the end, this/these biomarker(s) could serve as common biological endpoints bridging cells, animals, and patients.

### Limitations

In the present study, young animals were used. In addition to vincristine, which is used for hematological malignancies that can affect young patients, most of the anticancer drugs used here are prescribed in older adults for solid tumors. Various doses (both low and high), various treatment durations for each anticancer drug, and different time points for blood sampling could have been considered to emphasize the sensitivity and specificity of biomarker usefulness with respect to CIPN severity. Further non-clinical studies should be conducted on both male and female animals to improve the translationality of the results. Although CIPN is described as more severe in females than in male patients [3,65], the results in animals are less clear, with some sex differences being more pronounced in male animals than in female ones, and vice versa [66]. 

Regarding these study limitations with single doses, various treatment durations, single time points, and young male animals, these conditions and variabilities should have emphasized the robustness of the biomarkers assessed. Finally, it should be kept in mind that this pilot and exploratory study did not aim to describe precisely the kinetic, specificity, and sensitivity of each serum protein assessed. This pilot study aimed to screen a panel of serum biomarkers that might be of interest for further and specific studies on a limited number of biomarkers.

## 5. Conclusions

None of the assessed biomarkers can be recommended as a unique biomarker of CIPN-related pain, regardless of the specific anticancer drug used. Nevertheless, the results presented here highlight MCP-1, NfL, and RANTES as potential candidates, and further studies are needed to define their specificity, sensitivity, onset, and kinetics during the administration of anticancer drugs. Identifying blood biomarkers for peripheral neuropathy symptoms remains an important unmet need, not only in non-clinical and clinical studies but also for routine practice.

## Figures and Tables

**Table 1:** Serum concentrations of biomarkers for treated and control animals for oxaliplatin, paclitaxel, bortezomib, and vincristine. Results are presented by mean ± standard deviation. (EGF—Epidermal growth factor, G-CSF—Granulocyte-Colony Stimulating Factor, GFAP—Glial fibrillary acidic protein, GM-CSF—Granulocyte-Macrophage Colony-Stimulating Factor, GRO/KC—Growth-regulated α protein, IFNγ—Interferon gamma, IL-1α—Interleukin 1 alpha, IL-1β—Interleukin 1 beta, IL-2—Interleukin 2, IL-4—Interleukin 4, IL-5—Interleukin 5, IL-6—Interleukin 6, IL-10—Interleukin 10, IL-12(p70)—Interleukin 12, IL-13—Interleukin 13, IL-17A—Interleukin 17a, IL-18—Interleukin 18, IP-10—Interferon gamma-induced protein 10, LIX—Lipopolysaccharide-induced CXC chemokine, MCP-1—monocyte chemoattractant protein 1, MIP-1α—Macrophage inflammatory protein-1 alpha, MIP-2—Macrophage Inflammatory Protein-2, NfL—Neurofilament light chain, NGF—Nerve growth factor, OPN—Osteopontin, RANTES—Regulated upon Activation, Normal T Cell Expressed and Presumably Secreted, TNFα—Tumor necrosis factor alpha, and VEGF—Vascular endothelial growth factor).

Biomarkers (pg/mL)	Oxaliplatin		Paclitaxel		Bortezomib		Vincristine		Comparison of Controls
	Controls (n = 8)	Treated (n = 10)	Controls (n = 8)	Treated (n = 9)	Controls (n = 8)	Treated (n = 8)	Controls (n = 12)	Treated (n = 12)	
EGF	0.9 ± 0.0 ^#^	0.9 ± 0.0 ^#^	0.9 ± 0.0 ^#^	1.3 ± 1.1	0.9 ± 0.0 ^#^	0.9 ± 0.0 ^#^	0.9 ± 0.0 ^#^	0.9 ± 0.0 ^#^	NA
Eotaxin	8.1 ± 6.7	8.3 ± 6.3	18.9 ± 13.7	17.1 ± 18.4	11.5 ± 7.7	6.8 ± 6.2	7.6 ± 5.9	14.4 ± 12.7	NS
Fractalkine	81.3 ± 23.6	71.0 ± 19.2	90.5 ± 7.6	90.3 ± 24.8	81.0 ± 13.6	71.3 ± 24.8	72.5 ± 10.6	91.8 ± 13.3 **	§
G-CSF	6.7 ± 4.4	6.0 ± 4.7	21.7 ± 25.0	17.9 ± 30.7	6.2 ± 3.3	9.0 ± 12.2	6.5 ± 6.8	5.3 ± 2.5	NS
GFAP	15.6 ± 0.0 ^#^	15.6 ± 0.0 ^#^	15.6 ± 0.0 ^#^	15.6 ± 0.0 ^#^	15.6 ± 0.0 ^#^	15.6 ± 0.0 ^#^	15.6 ± 0.0 ^#^	15.6 ± 0.0 ^#^	NA
GM-CSF	10.0 ± 0.0 ^#^	10.0 ± 0.0 ^#^	10.0 ± 0.0 ^#^	10.0 ± 0.0 ^#^	10.0 ± 0.0 ^#^	10.0 ± 0.0 ^#^	10.0 ± 0.0 ^#^	10.0 ± 0.0 ^#^	NA
GRO/KC	58.2 ± 0.0 ^#^	58.2 ± 0.0 ^#^	58.2 ± 0.0 ^#^	58.2 ± 0.0 ^#^	58.2 ± 0.0 ^#^	58.2 ± 0.0 ^#^	58.2 ± 0.0 ^#^	58.2 ± 0.0 ^#^	NA
IFNγ	33 ± 64	44 ± 74	206 ± 284	186 ± 374	70 ± 101	57 ± 96	37 ± 92	29 ± 58	NS
IL-1α	49 ± 25	40 ± 0.0 ^#^	85 ± 82	93 ± 158	40 ± 0 ^#^	62 ± 64	40 ± 0 ^#^	40 ± 0 ^#^	§
IL-1β	20 ± 15	14 ± 9	47 ± 35	55 ± 38	22 ± 17	26 ± 15	34 ± 25	179 ± 305	NS
IL-2	49 ± 73	34 ± 51	114 ± 95	89 ± 139	53 ± 46	39 ± 49	20 ± 22	22 ± 18	NS
IL-4	35.8 ± 40.3	19.7 ± 4.9	57.7 ± 58.3	60.4 ± 98.4	26.6 ± 17.3	33.8 ± 38.2	22.0 ± 14.0	18.7 ± 2.4	NS
IL-5	122 ± 70	110 ± 64	212 ± 109	159 ± 145	113 ± 74	149 ± 78	113 ± 49	119 ± 37	NS
IL-6	282 ± 7	305 ± 80	452 ± 314	478 ± 593	280 ± 0 ^#^	280 ± 0 ^#^	280 ± 0 ^#^	280 ± 0 ^#^	§
IL-10	17 ± 19	8 ± 0.0 ^#^*	48 ± 46	38 ± 51	21 ± 21	12 ± 10	21 ± 22	106 ± 181	NS
IL-12(p70)	203 ± 171	122 ± 104	304 ± 262	351 ± 438	219 ± 159	272 ± 308	152 ± 93	92 ± 77	NS
IL-13	17.5 ± 0.0 ^#^	17.5 ± 0.0 ^#^	35.9 ± 27.6	31.9 ± 39.2	17.5 ± 0.0 ^#^	26.3 ± 24.7	17.5 ± 0.0 ^#^	17.5 ± 0.0 ^#^	§
IL-17A	17.7 ± 17.8	15.0 ± 10.5	57.5 ± 41.7	49.9 ± 70.2	22.7 ± 22.4	54.5 ± 92.2	16.6 ± 16.3	11.5 ± 12.1	§
IL-18	150 ± 71	121 ± 87	247 ± 117	192 ± 157	122 ± 60	129 ± 65	153 ± 81	353 ± 181 **	NS
IP-10	391 ± 113	435 ± 175	438 ± 118	488 ± 95	410 ± 107	378 ± 115	393 ± 99	585 ± 208 *	NS
Leptin	5964 ± 2515	5094 ± 2872	7720 ± 3797	8257 ± 2664	6602 ± 3301	4473 ± 1069	9962 ± 6639	7736 ± 5571	NS
LIX	3476 ± 768	4192 ± 1104	3808 ± 950	4833 ± 1679	4967 ± 1371	2722 ± 1081 **	5386 ± 1630	9536 ± 2792 **	§
MCP-1	633 ± 111	979 ± 294 *	819 ± 399	857 ± 415	768 ± 321	819 ± 222	819 ± 167	7575 ± 2457 ***	NS
MIP-1α	50 ± 15	46 ± 10	42 ± 11	42 ± 14	38 ± 10	35 ± 14	37 ± 10	288 ± 95 ***	NS
MIP-2	23.9 ± 0.0 ^#^	23.9 ± 0.0 ^#^	24.2 ± 0.7	28.1 ± 9.5	23.9 ± 0.0 ^#^	23.9 ± 0.0 ^#^	23.9 ± 0.0 ^#^	24.5 ± 1.9	NS
NfL	31.8 ± 9.6	22.0 ± 3.6 *	21.5 ± 11.5	27.5 ± 5.9 *	26.1 ± 8.3	24.6 ± 5.6	25.7 ± 9.0	75.0 ± 13.5 ***	NS
NGF	6.8 ± 4.2	5.3 ± 0.0 ^#^	6.3 ± 3.0	5.4 ± 0.2	5.3 ± 0.0 ^#^	5.3 ± 0.0 ^#^	5.3 ± 0.0 ^#^	5.3 ± 0.0 ^#^	NS
OPN	0.8 ± 0.1	0.9 ± 0.2	0.8 ± 0.2	0.8 ± 0.2	0.8 ± 0.1	0.8 ± 0.2	0.8 ± 0.1	0.9 ± 0.2 *	NS
RANTES	3008 ± 468	3887 ± 763 *	3016± 462	3060 ± 839	3181 ± 472	3061 ± 572	3036 ± 778	8394 ± 1318 ***	NS
TNFα	4.8 ± 3.7	6.0 ± 4.3	10.7 ± 9.3	12.0 ± 13.9	6.4 ± 3.9	6.1 ± 4.2	5.8 ± 2.3	6.1 ± 4.0	NS
VEGF	108 ± 21	127 ± 26	126 ± 12	127 ± 28	105 ± 12	98 ± 25	104 ± 10	227 ± 35 ***	§

^#^ Concentrations below the limit of quantification. NA: not applicable because of concentrations below the lower limit of quantification for all groups of animals. NS: not significant. § *p* < 0.05 for comparison of control animals (Kruskal–Wallis test). * *p* < 0.05, ** *p* < 0.01, and *** *p* < 0.001 for treated vs. control animals (Mann–Whitney test).

## Data Availability

Data will be available upon request to the corresponding author.

## Supplementary Materials

The following supporting information can be downloaded at: https://www.mdpi.com/article/10.3390/toxics11121004/s1, Table S1: Serum concentrations of biomarkers for treated and control animals for oxaliplatin, paclitaxel, bortezomib, and vincristine.
