# Supplementary material for: Exploring Serum Biomarkers for Neuropathic Pain in Rat Models of Chemotherapy-Induced Peripheral Neuropathy: A Comparative Pilot Study with Oxaliplatin, Paclitaxel, Bortezomib, and Vincristine
Source: Toxics. 2023 Dec 8;11(12):1004. doi: 10.3390/toxics11121004 (PMC10747971; doi:10.3390/toxics11121004)
Supplement: Supplementary file 1 [file toxics-11-01004-s001.zip › toxics-2725751-supplementary.pdf]

Supplementary file

Table S1. Technical characteristics of biomarker monitoring

Proteins	Function of proteins	Apparatus	Commercial kits	LLOQ (pg/mL)
EGF – Epidermal growth factor	Stimulates the growth of various epidermal and epithelial tissues	Bio-Plex® 200 System Bio-Rad Laboratories, Inc., USA	RECYMAG65K27PMX Merck®, France	0.9
Eotaxin – CCL11	Chemotaxis, Inflammatory response	Bio-Plex® 200 System Bio-Rad Laboratories, Inc., USA	RECYMAG65K27PMX Merck®, France	4.7
Fractalkine – CX3CL1	Cell adhesion, Chemotaxis, Host-virus interaction, Inflammatory response	Bio-Plex® 200 System Bio-Rad Laboratories, Inc., USA	RECYMAG65K27PMX Merck®, France	0.7
G-CSF – Granulocyte-Colony Stimulating Factor	growth and differentiation of granulocytes and the monocytes-macrophages	Bio-Plex® 200 System Bio-Rad Laboratories, Inc., USA	RECYMAG65K27PMX Merck®, France	4.5
GFAP – Glial fibrillary acidic protein	Cell-specific marker of astrocytes	ELISA microplate reader Biotek	orb439741 Biorbyt, Cambridgeshire, UK	15.6
GM-CSF – Granulocyte-Macrophage Colony-Stimulating Factor	Growth and differentiation of granulocytes, macrophages, eosinophils and erythrocytes	Bio-Plex® 200 System Bio-Rad Laboratories, Inc., USA	RECYMAG65K27PMX Merck®, France	10.0
GRO/KC – Growth regulated α protein, CXCL1	Inflammatory response	Bio-Plex® 200 System Bio-Rad Laboratories, Inc., USA	RECYMAG65K27PMX Merck®, France	58.2
IFNγ – Interferon gamma	Antiviral defense, Growth regulation	Bio-Plex® 200 System Bio-Rad Laboratories, Inc., USA	RECYMAG65K27PMX Merck®, France	10.5
IL-1α – Interleukin 1 alpha	Inflammatory response	Bio-Plex® 200 System Bio-Rad Laboratories, Inc., USA	RECYMAG65K27PMX Merck®, France	39.9
IL-1β – Interleukin 1 beta	Inflammatory response	Bio-Plex® 200 System Bio-Rad Laboratories, Inc., USA	RECYMAG65K27PMX Merck®, France	2.3
IL-2 – Interleukin 2	Adaptive immunity, Immunity	Bio-Plex® 200 System Bio-Rad Laboratories, Inc., USA	RECYMAG65K27PMX Merck®, France	9.9
IL-4 – Interleukin 4	B-cell activation	Bio-Plex® 200 System Bio-Rad Laboratories, Inc., USA	RECYMAG65K27PMX Merck®, France	17.9
IL-5 – Interleukin 5	Survival, differentiation, and chemotaxis of eosinophils	Bio-Plex® 200 System Bio-Rad Laboratories, Inc., USA	RECYMAG65K27PMX Merck®, France	19.8
IL-6 – Interleukin 6	Pro-inflammatory function (acute phase)	Bio-Plex® 200 System Bio-Rad Laboratories, Inc., USA	RECYMAG65K27PMX Merck®, France	279.9
IL-10 – Interleukin 10	Anti-inflammatory functions	Bio-Plex® 200 System Bio-Rad Laboratories, Inc., USA	RECYMAG65K27PMX Merck®, France	8.3
IL-12(p70) – Interleukin 12	Host-virus interaction	Bio-Plex® 200 System Bio-Rad Laboratories, Inc., USA	RECYMAG65K27PMX Merck®, France	40.3

IL-13 – Interleukin 13	Allergic inflammation and immune response to parasite infection	Bio-Plex® 200 System<br>Bio-Rad Laboratories, Inc., USA	RECYMAG65K27PMX<br>Merck®, France	17.5
IL-17A – Interleukin 17a	Adaptive immunity, Immunity, Inflammatory response, Innate immunity	Bio-Plex® 200 System<br>Bio-Rad Laboratories, Inc., USA	RECYMAG65K27PMX<br>Merck®, France	6.8
IL-18 – Interleukin 18	Inflammatory response	Bio-Plex® 200 System<br>Bio-Rad Laboratories, Inc., USA	RECYMAG65K27PMX<br>Merck®, France	6.2
IP-10 – Interferon gamma-induced protein 10, CXCL10	Chemotaxis, Inflammatory response	Bio-Plex® 200 System<br>Bio-Rad Laboratories, Inc., USA	RECYMAG65K27PMX<br>Merck®, France	1.4
Leptin	Regulation of energy balance<br>Pro-inflammatory function	Bio-Plex® 200 System<br>Bio-Rad Laboratories, Inc., USA	RECYMAG65K27PMX<br>Merck®, France	10.2
LIX – Lipopolysaccharide-induced CXC chemokine, CXCL5	Neutrophil activation	Bio-Plex® 200 System<br>Bio-Rad Laboratories, Inc., USA	RECYMAG65K27PMX<br>Merck®, France	20.9
MCP-1 - monocyte chemoattractant protein 1, CCL2	Chemotaxis, Inflammatory response	Bio-Plex® 200 System<br>Bio-Rad Laboratories, Inc., USA	RECYMAG65K27PMX<br>Merck®, France	9.0
MIP-1α – Macrophage inflammatory protein-1 alpha, CCL3	Chemotaxis, Inflammatory response	Bio-Plex® 200 System<br>Bio-Rad Laboratories, Inc., USA	RECYMAG65K27PMX<br>Merck®, France	0.8
MIP-2 – Macrophage Inflammatory Protein-2, CXCL2	Chemotaxis, Inflammatory response	Bio-Plex® 200 System<br>Bio-Rad Laboratories, Inc., USA	RECYMAG65K27PMX<br>Merck®, France	23.9
NfL – Neurofilament light chain	Maintenance of neuronal caliber	Simple Plex Ella®<br>ProteinSimple, USA	Rat NfL Kit<br>ProteinSimple, USA	2.7
NGF – Nerve growth factor	Development and maintenance of the sympathetic and sensory nervous systems	Bio-Plex® 200 System<br>Bio-Rad Laboratories, Inc., USA	EPX01A-32117-901<br>ThermoFisher Scientifique®, France	5.3
OPN – Osteopontin	Biomineralization, Cell adhesion	Bio-Plex® 200 System<br>Bio-Rad Laboratories, Inc., USA	RKTX1MAG-37K<br>Merck®, France	30.0
RANTES – Regulated upon Activation, Normal T Cell Expressed and Presumably Secreted, CCL5	Chemotaxis, Inflammatory response	Bio-Plex® 200 System<br>Bio-Rad Laboratories, Inc., USA	RECYMAG65K27PMX<br>Merck®, France	1.3
TNFα – Tumor necrosis factor alpha	Pro-inflammatory function	Bio-Plex® 200 System<br>Bio-Rad Laboratories, Inc., USA	RECYMAG65K27PMX<br>Merck®, France	2.3
VEGF – Vascular endothelial growth factor	Angiogenesis, Differentiation	Bio-Plex® 200 System<br>Bio-Rad Laboratories, Inc., USA	RECYMAG65K27PMX<br>Merck®, France	2.6

Function of proteins are reported from Human Protein Atlas (proteinatlas.org)

LLOQ: lower limit of quantification
